# Risk Factors for and Consequences of CAA in Three Community-Based Clinical-Pathologic Studies

**DOI:** 10.1093/geroni/igaf122.1008

**Published:** 2025-12-31

**Authors:** David Bennett

**Affiliations:** Rush University Medical Center, Chicago, Illinois, United States

## Abstract

Data come from the Religious Orders Study and Rush Memory and Aging Project (ROSMAP), and the Pathology, Alzheimer’s and Related Dementias Study (PARDoS). To date, CAA was characterized in 2100+ ROSMAP autopsies (mean age 89, only 3 under age 65, 69% female, 95% non-Latino white). Over the past decade, we found that both APOE4, APOE2, APOEC2, were associated with CAA. In eQTL analyses using snRNAseq, microglial APOE expression was associated with CAA independent of the APOE4 locus. Microglial ITGAX and SPPI expression were also associated with CAA. LDL cholesterol and chronic kidney disease were both associated with CAA. Controlling for other pathologies, CAA was associated with a lower level of cognition but not to rate of cognitive decline. Overall, the attributable risk of AD from CAA exceeded 8% and was greater than athero- and arteriolo-sclerosis accounting for other neurodegenerative diseases. In addition, CAA was associated with cerebral microbleeds and enlarged perivascular spaces (EPVS) in the temporal and occipital lobes. CAA was also associated with epilepsy and interestingly with risk aversion. In PARDoS, more than 4000 cases were characterized for CAA (mean age 76, 1 third <65, 48% female, 58% white). Adjusted for age, CAA did not differ by sex or race. CAA was present in 5% <65 with the youngest person <40, and a quarter 65+. CAA was associated with dementia adjusted for demographics but was attenuated and no longer significant adjusting for other neuropathologies. We identified several risk factors for and consequences of CAA.

